# Assessment of Psychological Implications and Quality of Life After Different Cardiac Device Implantation in Saudi Arabia

**DOI:** 10.7759/cureus.52338

**Published:** 2024-01-15

**Authors:** Naeem Alshoaibi, Alaa Aljazar, Maal Bushnag, Afnan Aseeri, Layan Baeesa, Shahad Alruwaithi, Roba Bashawri, Amjad Marouf, Refan Alsaeed

**Affiliations:** 1 College of Medicine, King Abdulaziz University, Jeddah, SAU

**Keywords:** saudi arabia, implant, cardiac device, quality of life, anxiety, depression, psychology

## Abstract

Background: Cardiac device therapy is likely to affect different aspects of patients' psychological well-being, such as their quality of life. The aim of this study was to examine the mental health status, specifically the conditions of depression and anxiety regarding implantable cardiac device patients.

Methods: A cross-sectional retrospective study was conducted in January 2022. This study was conducted at King Abdul-Aziz University Hospital in Jeddah, Saudi Arabia. All patients aged more than 18 years old who underwent heart device implantation for six months or more formed the study population. A total of 30 implantable cardioverter defibrillator (ICD) subtypes were used in our patients (45.8%), including cardiac resynchronization therapy device (CRT-D) in seven patients (14.6%) and one subcutaneous ICD (2.1%). A pacemaker was used in 18 patients (37.5%). Binary logistic regression analysis was conducted to identify the association between type of cardiac implantation device and the likelihood of having abnormal depression and anxiety score.

Results: A total of 48 patients participated in this study. Hypertension was the most frequently associated risk factor in our sample (64.6%; n=31). In comparison between ICD users and pacemaker users in terms of the SF-36 general health survey, a marginally significant difference was noted in the role of limitations due to emotional health (63 ± 28.6) for ICD patients compared to pacemaker patients (81.8 ± 28.1), (p=0.050). However, pacemaker patients showed a significant favourable social functioning score (90.1 ± 17.7) compared to ICD patients (71.5 ± 19), (p=0.001). There is no significant difference noted regarding the other domains. Binary logistic regression analysis identified that patients who are using ICD were seven times more likely to have abnormal anxiety score (odds ratio: 7.00 (95% confidence interval: 1.36-35.9) (p=0.020).

Conclusion: This study identified a potential association between cardiac devices and the anxiety and quality of life of patients. Nonetheless, further investigation is warranted to assess the psychological and physiological effects of cardiac device therapy on patients, in addition to examining the effects of implantation and follow-up on cardiac function and cardiac symptoms.

## Introduction

Depression is a prevalent psychiatric condition distinguished by persistent negative thinking patterns and an inability to derive pleasure from previously enjoyable experiences. An episode can be categorized into three distinct levels: mild, moderate, or severe. The incidence of depression exhibits significant variability, contingent upon several characteristics such as gender and age. Depression is more prevalent among females, reaching its highest incidence between the ages of 55 and 74. However, it can also manifest in both children and adults [1-4]. Over 350 million individuals have been diagnosed with depression [5]. Depression is influenced by various factors, including psychological, social, and biological characteristics, as well as adverse life events [6]. Depression and physical health are strongly correlated, as untreated chronic diseases are often worsened by unrecognized depression, leading to unfavorable outcomes. Depression and cardiovascular illnesses are interconnected, occurring simultaneously and serving as mutual risk factors. The prevalence of depression in cardiovascular patients ranges from 20% to 45%, and it directly affects various aspects of the cardiovascular system, such as heart rate, blood pressure, vascular resistance, vasomotor tone, plasma volume, and blood viscosity [7]. According to the guidelines, patients with cardiovascular issues need to have cardiac devices implanted based on their specific condition. The devices encompass ventricular single-chamber pacing (VVI) pacemakers, dual chamber pacemaker (DDD) pacemakers, leadless pacemakers, cardiac resynchronization therapy (CRT)-P devices, CRT-D devices, implantable cardioverter defibrillators (ICDs), and subcutaneous ICDs.

When the device is implanted in response to sudden cardiac death or life-threatening arrhythmias, the patient frequently experiences severe psychological distress. Furthermore, individuals who are undergoing primary prevention with a device are required to have a device implanted into their body, which alters the rhythm and function of their hearts [8]. In addition, cardiac device therapy is associated with persistent complications, including intracardiac lead failure and infections [9,10]. In this regard, it is probable that cardiac device therapy will have an impact on various dimensions of psychological well-being, including quality of life. However, in practice, these aspects are frequently overlooked, inadequately diagnosed, and undertreated [11-13]. Previous studies examined the psychological aspects of different disease areas and different study populations in the Middle East [14-19], with limited studies that examined the psychological implications of cardiac device implantation. Therefore, this study was conducted to evaluate the mental health status, specifically the conditions of depression and anxiety regarding implantable cardiac device patients.

## Materials and methods

Study design

A cross-sectional retrospective study was conducted in January 2022. Patients who underwent heart device implantation for six months or more were identified. Eligible patients attending physician appointments were identified by the researchers with the aid of clinic receptionists. Patients were surveyed using the study tools and then their medical history were extracted using their electronic medical records at the hospital. Patients were recruited using convenience sampling technique.

Study setting

This study was conducted at King Abdul-Aziz University Hospital in Jeddah, Saudi Arabia. King Abdulaziz University Hospital is the first teaching hospital in the Kingdom of Saudi Arabia. It offers exceptional medical services in all aspects of ophthalmology, ear, nose, and throat medicine, facilitated by proficient and knowledgeable consultants, physicians, and technicians who utilize the most advanced technical resources and infrastructure in the kingdom. Secondary and tertiary health care services are offered by the hospital, with an emphasis on ophthalmology, ear, nose, and throat. However, internal medicine, surgery, pediatrics, obstetrics and gynecology, anesthesia, psychiatry, X-rays, medical rehabilitation, laboratory medicine, and dental services are also provided as primary health care specialties.

Study population

All patients aged more than 18 years old who underwent heart device implantation for six months or more formed the study population. Any patient on antidepressants for a previous diagnosis of depression or suffering from predisposed mental illness, patients who suffered from focal neurological lesions, and patients who refused to participate were excluded.

Data collection

Data for this study was collected using a previously developed questionnaire tool and from patients' medical records. Data included the patients' demographics, risk factors, physical health, type of implanted device, complications of the implanted device, types of heart diseases, and medication use history. In addition, device-related assessment on both physical and mental aspects, the Hospital Anxiety and Depression Scale (HADS) scale for depression and anxiety assessment, and the SF-30 questionnaire for the assessment of the quality of life. HADS is employed to assess levels of anxiety and depression among patients in a broad medical community [14,20]. This questionnaire consists of seven questions pertaining to anxiety and seven questions pertaining to depression. The HADS questionnaire is administered to patients in a secondary-care context to assess the existence and severity of depressed and anxious symptoms. The SF-36 questionnaire, consisting of 36 items, is widely used to assess Health-Related Quality of Life [21]. The SF-36 assesses eight dimensions: physical functioning (PF), role physical (RP), bodily pain (BP), social functioning (SF), role emotional (RE), general health (GH), vitality (VT), and mental health (MH).

Ethical approval

The ethical approval for this study was obtained from the Unit of Biomedical Ethics Research Committee of King Abdulaziz University, Jeddah, Saudi Arabia, wherein they reviewed and approved this study (Reference No.421/21). Informed consent was obtained from all participants.

Statistical analysis

The Statistical Package for Social Science Software (SPSS), version 29 (IBM Corp., Armonk, NY, USA) was used to analyse the data for this study. Categorical variables were presented using frequencies and percentages. Continuous variables were presented using the mean and standard deviation (SD) due to the normality of the data. A Chi-squared test and Fisher’s test were used to compare proportions for categorical variables. Binary logistic regression analysis was conducted to identify the association between type of cardiac implantation device and the likelihood of having abnormal depression and anxiety scores. In addition, multiple logistic regression analysis was conducted in order to address potential confounding variables. The statistically significant level was assigned as p-value less than 0.05.

## Results

Baseline characteristics of the study participants

A total of 48 patients participated in this study. The vast majority of participants were Arab (81.3%; n=39), Asians presented 10.4% of the sample (n=5), and 8.3% (n=4) were African. Type one heart failure class of New York Heart Association (NYHA) was found in 27 patients (56.3%). A total of 30 ICD subtypes were used in our patients (45.8%), including CRT-D in seven patients (14.6%) and one subcutaneous ICD (2.1%). A pacemaker was used in 18 patients (37.5%); three of them used the leadless pacemaker type (6.3%). Regarding the complications of devices, shocked ICD was reported in 10 patients (20.8%), lead dislodgement in six patients (12.5%), and one case (2.1%) for each of pneumothorax and diaphragmatic stimulation (Table 1).

**Table 1:** Baseline characteristics of the study participants. NYHA: The New York Heart Association Functional Classification; CRT-D: Cardiac resynchronization therapy device; DDD: Dual chamber pacemaker; ICD: Implantable cardioverter defibrillator

Variable	Frequency	Percentage	
Ethnicity:			
Arab	39		81.3%
Asian	5		10.4%
African	4		8.3%
NYHA class:			
1	27		56.3%
2	13		27.1%
3	6		12.5%
4	2		4.2%
Ankle swelling	17		35.4%
Types of devices:			
ICD	22		45.8%
DDD (pacemaker)	15		31.3%
CRT-D	7		14.6%
Leadless Pacemaker	3		6.3%
Subcutaneous ICD	1		2.1%
Complications:			
Shocked ICD	10		20.8%
Lead dislodgement	6		12.5%
Pneumothorax	1		2.1%
Diaphragmatic stimulation	1		2.1%

Distribution of risk factors among the patients

**Figure 1:**
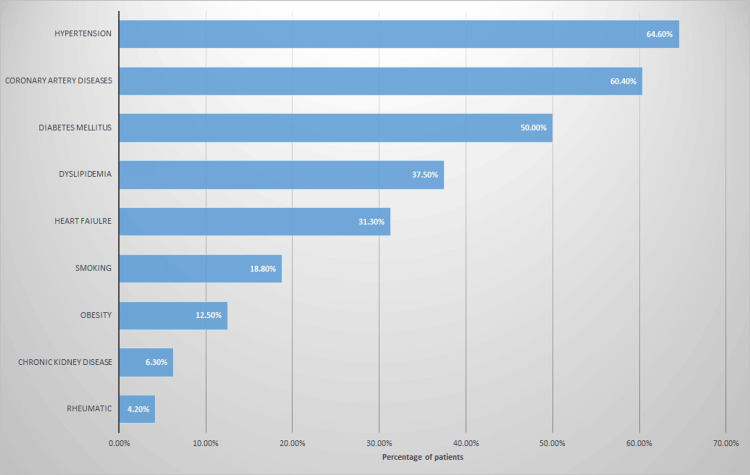
The prevalence of risk factors among the patients.

Figure 1 below summarizes the associated risk factors in our patients. Hypertension was the most frequently associated risk factor in our sample (64.6%; n=31), followed by coronary artery disease (CAD) (60.4%; n=29), then diabetes mellitus (50%; n=24), and dyslipidemia (37.5%; n=18).

Medication utilization profile of the patients

**Figure 2:**
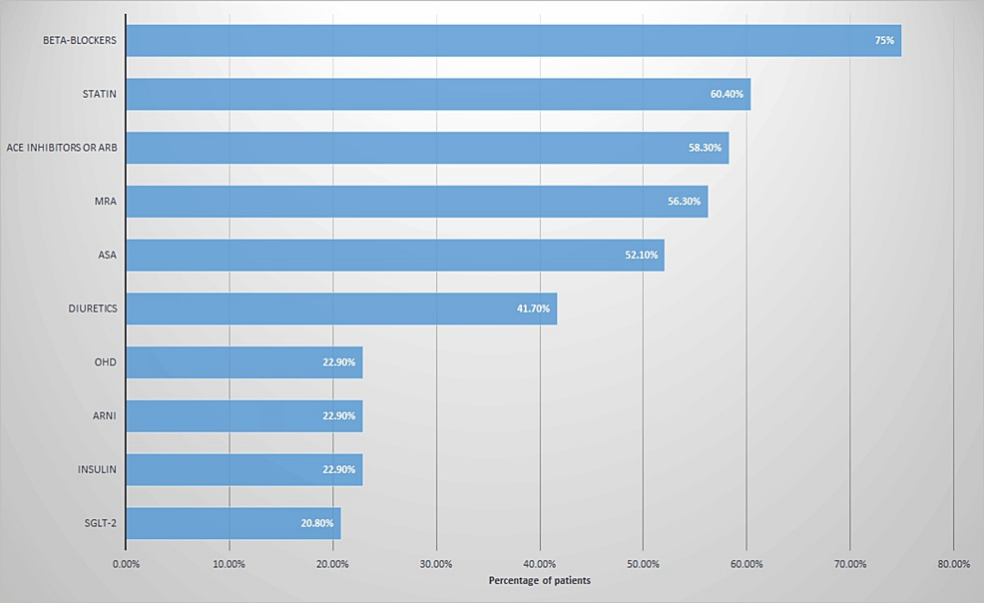
Medications use history of the patients. SGLT2 inhibitors: Sodium-Glucose Transport Protein 2 (SGLT2) Inhibitors; ARNI: Angiotensin receptor-neprilysin inhibitor; OHD: Oral hypoglycemic agents; ASA: Acetylsalicylic acid; MRA: Mineralocorticoid receptor antagonist; ACE: Angiotensin-converting enzyme inhibitors; ARB: Angiotensin II receptor blockers

Figure 2 shows medication use history of the patients. Beta-blocker drugs were the most frequently concurrently used drug in our patients (75%; n=36), followed by statins (60.4%; n=29), then angiotensin-converting enzyme (ACE) inhibitors or angiotensin receptor blockers (ARBs) (58.3%; n=28), and mineralocorticoid receptor antagonist (MRA) and acetylsalicylic acid (ASA) (56.3%; n=27).

Patients’ quality of life

Table 2 summarizes the mean scores of the SF36-general health survey. In comparison between ICD users and pacemaker users in terms of the SF-36 general health survey, a marginally significant difference was noted in the role of limitations due to emotional health (63 ± 28.6) for ICD patients compared to pacemaker patients (81.8 ± 28.1) (p=0.050). However, pacemaker patients showed a significant favourable social functioning score (90.1 ± 17.7) compared to ICD patients (71.5 ± 19) (p=0.001). There is no significant difference noted regarding the other domains.

**Table 2:** The mean scores of the SF36-general health survey. ICD: Implantable cardioverter defibrillator; SD: Standard deviation

Item	All patients		ICD patients		Pacemaker patients		P-value
Domain	Mean	SD	Mean	SD	Mean	SD	
1- Physical functioning	67.5	± 21.1	63.0	± 21.1	75.0	± 19.3	0.074
2- Role physical	57.4	± 31.5	52.1	± 29.2	66.2	± 34.1	0.155
3- Role emotional	70.6	± 29.5	63.9	± 28.6	81.8	± 28.1	0.050
4- Vitality	67.9	± 16.6	69.0	± 16.4	65.9	± 17.1	0.923
5- Mental health	82.5	± 16.4	79.5	± 17.9	87.7	± 12.4	0.080
6- Social functioning	78.5	± 20.5	71.5	± 19.0	90.1	± 17.7	0.001
7- Bodily pain	63.6	± 30.8	60.1	± 28.2	69.3	± 34.8	0.206
8- General health	69.2	± 17.3	71.1	± 16.8	66.1	± 18.1	0.385

Patients’ anxiety and depression profile

Borderline abnormal HADS total score for depression were reported by 10 patients in the ICD group (33.3%) compared to two patients in the pacemaker group (11.1%), yet this difference was not statistically significant (p=0.085). However, a statistically significant difference was found in HADS total scores for anxiety between both groups, that ICD showed higher frequencies of abnormal and borderline abnormal scores, 13.3% (abnormal) and 33.3% (borderline abnormal), compared to 0.0% and 11.1% in the pacemaker group, respectively (p=0.034) (Table 3).

**Table 3:** HADS total score classification of the patients ICD: Implantable cardioverter defibrillator; HADS: Hospital Anxiety and Depression Scale

HADS total score classification	All patients			ICD patients		Pacemaker patients		P-value
	Frequency	Percentage		Frequency	Percentage	Frequency	Percentage	
Depression								
Borderline abnormal	12	25.0%	10		33.3%	2	11.1%	0.085
Normal	36	75.0%	20		66.7%	16	88.9%	
Anxiety								
Abnormal	4	8.3%	4		13.3%	0	0.0%	0.034
Borderline abnormal	12	25%	10		33.3%	2	11.1%	
Normal	32	66.7%	16		53.3%	16	88.9%	

Binary logistic regression analysis found that there is no statistically significant association between the type of device and the development of depression (odds ratio: 4.00 (95% confidence interval: 0.77-20.92) (p=0.101). Similarly, multiple logistic regression analysis model (adjusting for NYHA class) confirmed the same findings (odds ratio: 0.93 (95% confidence interval: 0.11-7.99) (p=0.949). On the other hand, binary logistic regression analysis identified that patients who are using ICD were seven times more likely to have abnormal anxiety score (odds ratio: 7.00 (95% confidence interval: 1.36-35.9) (p=0.020). However, multiple logistic regression analysis model (adjusting for NYHA class) identified that there is no statistically significant difference between the patients who are using ICD compared to those who are using pacemakers in terms of their anxiety level (odds ratio: 3.47 (95% confidence interval: 0.58-20.79) (p=0.173).

## Discussion

The aim of this study was to evaluate the mental health status, specifically the conditions of depression and anxiety regarding implantable cardiac device patients. It is probable that cardiac device therapy will have an impact on various dimensions of patients' psychological well-being, including quality of life [22].

In our study, we identified lower quality of life score in the role of limitation due to emotional health domain among ICD patients (63 ± 28.6) compared to pacemaker patients (81.8 ± 28.1) (p=0.050). However, pacemaker patients showed a significant favourable social functioning score (90.1 ± 17.7) compared to ICD patients (71.5 ± 19) (p=0.001). In line with our study findings, a previous systematic review assessed the quality of life linked to implantable cardiac devices [22]. This systematic review found that the increase in quality of life was significant for patients with pacemakers and life-threatening ventricular arrhythmias with shock deliveries to heart assist devices (left ventricular assist device (LVAD)). However, the improvement was less pronounced for patients with ICD [22]. An assist device effectively alleviates symptoms by efficiently assuming the heart pump function, despite the inherent risks and physical demands of the implantation procedure. Hence, the beneficial impact of pacemakers can be attributed to the enhancement of physical symptomatology after the implantation [22]. The primary reasons for implantation include the presence of evident symptomatic bradycardia resulting in fainting, symptoms of chronotropic incompetence, or the need for rhythm control therapy in patients experiencing symptomatic supraventricular tachycardia [22]. The ICD is the only device that does not immediately improve physical symptoms. Instead, it serves as a preventive tool to safeguard patients against sudden cardiac death. This could also account for the reason why the ICD was correlated with a lesser degree of improvement in quality of life compared to the other two implantable devices [22].

According to a previous comprehensive meta-analysis, it is predicted that over 20% of individuals with ICDs experience anxiety and depressive symptoms [11]. Pedersen et al. found that the selection of the ICD system, whether it be transvenous or subcutaneous, had no impact on the quality of life in ICD patients. Furthermore, both types of devices resulted in better quality of life [23]. Patients with pacemakers had comparable levels, which correlated with an elevated incidence of physical and mental exhaustion following the implantation procedure [24,25]. Other research suggests that the use of leadless pacemakers, a new technology, may offer a higher quality of life compared to conventional transvenous pacemakers [26]. The heightened prevalence of mental illness in individuals with heart conditions emphasize the importance of evaluating and addressing their quality of life [27,28]. The relationship between mental health and cardiovascular disease is reciprocal, since mental health disorders also have a negative impact on cardiovascular morbidity and mortality. This highlights the crucial significance of diagnosing and treating these conditions [29,30].

In our study, binary logistic regression analysis identified that patients who are using ICD were seven times more likely to have abnormal anxiety score (odds ratio: 7.00 (95% confidence interval: 1.36-35.9) (p=0.020). However, multiple logistic regression analysis model (adjusting for NYHA class) identified that there is no statistically significant difference between the patients who are using ICD compared to those who are using pacemakers in terms of their anxiety level (odds ratio: 3.47 (95% confidence interval: 0.58-20.79) (p=0.173). ICDs are intended to elicit electrical impulses to the heart in the event that an arrhythmia that poses a risk to life is identified [31]. Although these shocks have the potential to preserve lives, they can also induce significant distress and anxiety in the patients. Additionally, patients with ICDs frequently have more severe underlying cardiac conditions, and a higher perceived risk of mortality may be associated with the presence of an ICD [31,32].

Study limitations

The present study is not without its limitations. The cross-sectional design imposed limitations on our capacity to investigate causal relationships among the variables under investigation. A further potential limitation of this study is sampling bias, as it was conducted using a convenient sampling method, which could incorporate subjectivity into the findings. Additionally, the small sample size is a constraint. Expanding the sample size would enhance the reliability and applicability of the findings. The results of our study should therefore be interpreted with caution.

## Conclusions

This study revealed that ICD patients have more anxiety than pacemaker patients. Yet, we need more research measuring measures of quality of life as well as cardiac function and symptoms during implantation and follow-up to better understand how cardiac device therapy impacts patients’ quality of life and patients' psychology.
